# Risk factors for natural menopause before the age of 45: evidence from two British population-based birth cohort studies

**DOI:** 10.1186/s12905-022-02021-4

**Published:** 2022-11-08

**Authors:** Darina Peycheva, Alice Sullivan, Rebecca Hardy, Alex Bryson, Gabriella Conti, George Ploubidis

**Affiliations:** 1 Social Research Institute, University College London, London, UK; 2 Great Ormond Street Institute Of Child Health, University College London, London, UK; 3 School of Sport, Exercise and Health Sciences, Loughborough University, Loughborough, UK; 4 Department of Economics, University College London, London, UK

**Keywords:** Early menopause, Risk factors, Life course, Birth cohort, Social class, Breastfeeding, Cognition, Smoking, Alcohol, Exercise, Gynaecological problems

## Abstract

**Background:**

Menopause that occurs before the age of 45 and is not medically induced (referred to here as ‘early natural menopause’) affects around one in 10 women and has serious health consequences. These consequences include increased risk of all-cause mortality, cardiovascular disease, osteoporosis, and type 2 diabetes.

**Methods:**

We investigate risk factors for the onset of natural menopause before the age of 45 in two population-based prospective cohort studies in Britain: the 1958 cohort following 8959 women and the 1970 cohort following 8655 women. These studies follow women from birth to adulthood, and we use harmonized data on birth and early life characteristics, reproductive health, health behaviour, and socioeconomic characteristics for 6805 women who were pre-menopausal, peri-menopausal or had undergone natural menopause. Of these 6805 women, 3614 participated in the 1958 cohort (of which 368 had early menopause) and 3191 participated in the 1970 cohort (of which 206 had early menopause). Taking a life course approach, we focus on three distinct life stages - birth/early life, childhood, and early adulthood - to understand when risk factors are most harmful. Respecting the temporal sequence of exposures, we use a series of multivariable logistic regression models to estimate associations between early menopause and each potential risk factor adjusted for confounders.

**Results:**

We find that early menopause is influenced by circumstances at birth. Women born in lower social class families, whose mother smoked during the pregnancy or who were breastfed 1 month or less were more likely to undergo early menopause. Early menopause is also associated with poorer cognitive ability and smoking in childhood. Adult health behaviour also matters. Smoking is positively correlated with early menopause, while regular exercise and moderate frequency of alcohol drinking in women’s early thirties are associated with reduced risk of early menopause. The occurrence of gynaecological problems by women’s early thirties is also linked to early menopause.

**Conclusions:**

We demonstrate that characteristics at different periods of life are associated with early menopause. Some of these associations relate to modifiable behaviours and thus the risks of early menopause and the adverse health outcomes associated with it may be preventable.

**Supplementary Information:**

The online version contains supplementary material available at 10.1186/s12905-022-02021-4.

## Background

Menopause marks the permanent cessation of menstruation and the end of a woman’s reproductive life following alterations in the ovarian follicular function and the hypothalamic-pituitary function [1–3]. It may occur naturally (spontaneously) or be medically induced (e.g., surgical removal of both ovaries). Natural menopause, defined after 12 consecutive months of amenorrhea for which there is not obvious pathological or physiological cause, usually occurs between 45 and 55 years of age with an average age estimated between 50 and 52 years though there are slight variations between ethnic groups [4, 5]. Between 1 and 3% experience menopause without obvious cause before the age of 40, and between 5 and 10% experience it between the ages of 40 and 44. Here, we will follow the convention of referring to the onset of natural menopause before age 45 as *early menopause*^1^ [5, 6]. Earlier menopause is considered a marker of adverse health later in life [7]. Results from population-based samples have shown that women who experience early natural menopause have an increased risk of cardiovascular disease [8, 9], osteoporosis [10], type 2 diabetes [11], premature decline in cognitive function [12], decreased life expectancy and increased all-cause mortality [5, 13].

Past research suggests that the onset of natural menopause is strongly influenced by genetics [14, 15], but non-genetic factors can also play a role [16, 17]. There is, however, little consensus on the influence of non-genetic factors on the timing of natural menopause. Cigarette smoking has consistently been associated with early menopause, but the influence of several other factors such as reproductive health, health behaviour (e.g., alcohol consumption, exercise), and socioeconomic circumstances remains unclear. Consequently, it is critically important to establish the non-genetic factors associated with early natural menopause, especially those that are modifiable so that preventive health strategies can be considered.

The use of a life course approach for studying the timing of menopause is supported by the literature [18–20]. The life course approach enables identification of critical or sensitive periods during life where exposure to a particular risk factor is most harmful and may thus provide information on timely interventions [21]. A life course approach is useful for investigating age at menopause because a woman’s ovarian reserve declines gradually from about several million follicles post-conception to about 1 million at birth, 500,000 at menarche, and around a thousand prior to menopause [22]. This indicates that factors operating both before and after birth may play a role in follicle pool depletion and identifying such factors may help in understanding the biological process of menopause. Previous studies have noted that fetal and early life factors such as fetal growth, early life nutrition, early life stress and cognition, and childhood socioeconomic circumstances may influence the timing of menopause, but few studies have prospectively collected measures of multiple factors from early life alongside later life exposures [20, 23].

Previous research has suggested that restricted fetal growth, generally marked by low birthweight, may adversely affect the peak number of ovarian follicles and thus affect the timing of the menopause [5]. Several studies investigated the relationship between low birth weight and early menopause [23–29], but only few found a significant association [24–27]. In one of the few studies with information on gestational age, Tom et al. (2010) illustrated an increased risk for menopause before 45 for women with heavy weight at birth and high birthweight standardized by gestational age - a marker of faster fetal growth rate – indicating that growth rate rather than prematurity might be more important. Some studies showed a significant association with early menopause of low weight at 1 year [29] or at 2 years [23].

Poor nutrition has also been linked to the timing of menopause, with research showing that children who were not breastfed or breastfed for short periods, who had a low nutrient intake and slow post-natal growth may undergo menopause earlier [13, 30]. Breastfeeding was found to delay menopause in the 1946 National Survey of Health and Development (NSHD) [23, 31], but no association was found in UK Biobank (2016).

It has been hypothesised that prenatal exposure to cigarette smoke may affect the follicle pool by altering the development of ovarian follicles [32]. A negative effect of maternal smoking around birth on early menopause was observed in the UK Biobank [25] and a US cohort study of prenatal diethylstilbestrol exposure [33], while other studies found no association [26, 34, 35].

It is hypothesised that both young and old maternal age at birth may increase the risk of earlier menopause in the daughter [23]. If the maternal cycle is irregular at conception the maturational state of the oocyte at ovulation may not be optimal, which increases the risk of ovarian maldevelopment, and thus infertility and early menopause in the daughter [36]. However, Steiner et al. (2010) showed that having a mother aged 35 years or older at birth was associated with later age at menopause compared with mothers aged 20–34 years.

Several studies have indicated an association between disadvantaged childhood socioeconomic position and earlier menopause, and the impact of socioeconomic disadvantage has been considered to act through the effects of hardship experience throughout life and health behaviours [20, 37, 38]. Others have found no or weak associations [29, 39]. Socioeconomic position in childhood has been found to be more strongly related to early menopause than adult socioeconomic position in the NSHD [20, 37], while adverse lifetime socioeconomic position was associated with earlier menopause in the British Women’s Heart and Health Study [38]. Adult social class was not related to early menopause in a study using 1958 National Child Development Study (NCDS) data (up until age 45) [27].

Psychological health in early life has been linked to the onset of menopause, potentially acting though the function of the hypothalamic-pituitary-adrenal axis, which affects the reproduction function [31]. For example, parental separation, hypothesised as an indicator of early emotional stress, has been associated with earlier menopause in the NSHD [20].

Several studies have found that lower cognitive ability in childhood is associated with early menopause with earlier measures of cognitive ability being more strongly related to age at menopause than tests taken later in life [31, 39, 40]. It has been hypothesised that this relationship may be due to ovarian steroids from early in life which act to affect both neuron and ovarian follicle loss [23, 41]. However, heritability studies suggested potential “genetic programming”, raising the prospect that menopause and cognitive function may be influenced by the same genes [15, 17].

It has been hypothesized that fewer ovulatory cycles, crudely estimated by events contributing to a lower number of menstrual cycles, such as late age at menarche, longer menstrual cycles, and a higher number of full-term pregnancies, as well as length of use of oral contraceptives where menstrual cycles are likely anovulatory, result in later age at menopause [42]. The evidence, however, is contested [13, 25, 35–38], and it should be noted that the true number of ovulatory cycles cannot be determined accurately as cycles can be anovulatory (the release of an egg does not occur) even when menstrual bleeding occurs, with anovulatory cycles becoming more common with increasing age [43].

Gynaecological problems are another potential risk factor for early menopause. UK Biobank data showed no evidence of an association between such problems and early menopause [25]. However, several studies investigating the impact of pelvic or ovarian infections, or routine gynaecological surgeries, on fertility and ovarian function (primarily by studying response to ovarian stimulation during IVF cycles) have shown that women who do not respond to hormonal stimulation are more likely to experience early menopause [44–48].

Behavioural factors such as smoking, drinking and exercise affect endocrine function and women’s reproductive hormones, and thus may have an impact on the timing of menopause [49–52]. It has been consistently shown that women who smoke are more likely to experience menopause between 1 and 2 years earlier than non-smokers [13, 53–56]. Pooled analysis of 17 studies demonstrated that a higher number of cigarettes smoked per day and a longer period of cigarette smoking, including an earlier age at starting, were associated with early menopause [57]. While several studies have postulated that moderate consumption of alcohol delays menopause [58–60] by inducing oestrogen production [50, 51], others have shown associations with younger age at menopause [61] or found no relationship [62]. Moderate physical activity has been associated with later age at natural menopause [55], but other studies observed no association for either adolescent or adulthood physical activity [63].

Associations between body mass index (BMI) and obesity with ovarian reserve and menopause have been inconsistent and the mechanisms underlying the associations unclear [53, 64–68]. However, many studies used only a midlife BMI measure, whereas the effects of BMI may depend on the stage of menopause transition [69]. Alternatively, it may be that change in weight rather than weight at a particular point impacts the timing of menopause [70].

It has been suggested that the lack of consensus on the factors affecting the timing of natural menopause could be explained by methodological differences, e.g., differences in study populations and designs, differences in the definition of menopause, and varying use of controls in the analyses [4, 13]. Analysis of large-scale prospective studies can help resolve this lack of consensus [13].

We contribute to this literature with a study investigating non-genetic risk factors from across the life course for early natural menopause using data from two population-based prospective cohort studies of British women born in 1958 and 1970 and followed from birth through to childhood and adulthood. Using data from two studies increases the numbers experiencing menopause before the age of 45 which provides greater statistical power while also allowing assessment of consistency of associations across generations. Taking a life course approach, we investigate whether early life exposures (maternal smoking in pregnancy, maternal age at birth, birthweight, gestational age, and breastfeeding), reproductive characteristics (age at menarche, gynaecological problems, contraceptive use, and live births), health behaviour (smoking, alcohol consumption, and exercise), socioeconomic characteristics (father’s social class at birth and adult social class), cognitive ability, psychological distress and BMI are associated with early menopause. Respecting the timing and temporal order of exposures, we investigate the role of exposures during potentially critical or sensitive periods in early life alongside adult factors to understand the stages of life where exposure is important. There is substantial literature that the developmental origins of health lie in the early life period [71–73], and a life course approach is important not only for the development of health preventive strategies but for identifying the appropriate time to intervene [18].

## Methods

This study investigates non-genetic risk factors for early natural menopause at three distinct stages of life preceding the menopause onset – birth and early life, childhood, and early adulthood. We address the following research questions:

1. What birth and early life characteristics are associated with menopause before 45 years of age?
2. What childhood characteristics are associated with early menopause, while factors at birth are accounted for?
3. And lastly, what adult characteristics influence early menopause, while early life and childhood factors are accounted for?

The data used in this study is harmonized pooled individual level data comprising women from the 1958 National Child Development Study (NCDS) and the 1970 British Cohort Study (BCS70). The NCDS and BCS70 are prospective birth cohort studies that follow the lives of 8959 women (18,558 people) and 8655 women (18,037 people), respectively, born in England, Scotland, and Wales in a single week of March 1958 and March 1970 [74, 75]. Since birth, study members of the 1958 cohort (and/or their parents) have been interviewed 10 times at ages 7, 11, 16, 23, 33, 42, 44/45, 46, 50 and 55. We use information collected at birth and ages 7, 11, 16, 33, 42, 44/45 and 50 [76]. Since birth, study members of the 1970 cohort (and/or their parents) have been interviewed 9 times at ages 5, 10, 16, 26, 30, 34, 38, 42 and 46. We use information collected at birth and ages 5, 10, 16, 30, 38, 42 and 46 [77]. Participation in both studies is voluntary and requires verbal consent [78, 79].

### Menopause status

**Fig. 1:**
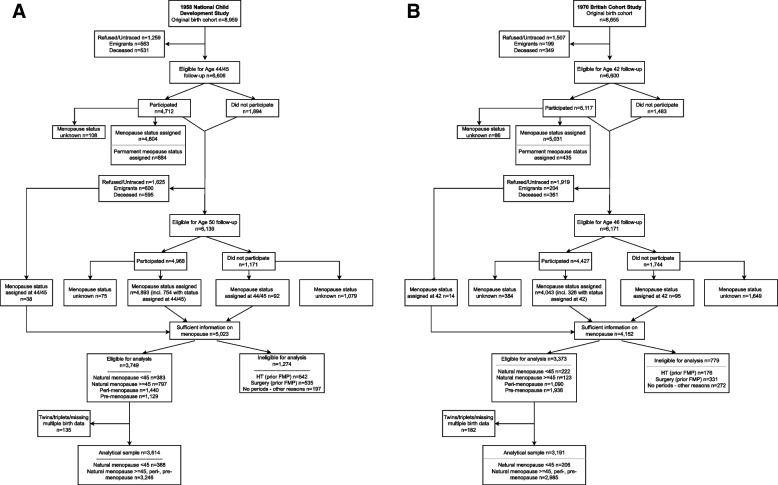
Participation and attrition in the 1958 National Child Development Study (**A**) and 1970 British Cohort Study (**B**)

Menopause status is determined using information collected at age 42 and 46 in BCS70 and age 44/45 and 50 in NCDS. Of the 8655 women in the 1970 cohort at birth, 5117 participated at the age 42 follow-up survey (of 6600 eligible for interview) and 4427 participated at the subsequent age 46 survey (of 6171 eligible). Of the 8959 women in the 1958 cohort at birth, 4712 participated at the age 44/45 follow-up survey (of 6606 eligible for interview) and 4968 women took part at the subsequent age 50 survey (of 6139 eligible) (Fig. 1A, B).

Women in both cohorts were asked if they had menstrual periods in the past 12 months. In the presence of amenorrhea, they were asked if they had periods in the past 3 months. Women with no periods in the past 12 months were asked about their age and the month at their final menstrual period (FMP) and the reason for amenorrhea. All women were asked about changes in the regularity of their menstrual periods in the last few years or before their FMP, about having undergone hysterectomy or bilateral oophorectomy, and hormone therapy (HT) use, including the dates of surgery or initiation of HT, and FMP prior to initiation of HT if menstruation has ceased before the HT start.

The derivation of menopause status followed widely accepted classification [4, 22]. Natural menopause was defined as 12 or more consecutive months of amenorrhea for which there was no obvious reason. Peri-menopausal women were those with 3 to 11 months of amenorrhea or whose periods became less regular in the absence of amenorrhea. Pre-menopausal women reported menstruation within the last 3 months. Those who have undergone hysterectomy or bilateral oophorectomy prior to their FMP or whose periods stopped for other obvious reasons (e.g., pregnancy, contraceptives, chemotherapy, or radiotherapy), as well as women who started HT prior to their FMP, were classified in separate categories. The same rules applied for the initial and follow-up sweep. However, once a woman had gone through natural menopause or a surgery (hysterectomy/bilateral oophorectomy) or had initiated HT prior to the FMP, their menopausal status remained unchanged for the subsequent survey. Four thousand one hundred fifty-two women in the 1970 cohort and 5023 women in the 1958 cohort provided sufficient information for menopause status (by age 46 in the 1970 and by age 50 in the 1958 cohort) to be assigned. Only pre-, peri-menopausal and women with natural menopause were included in this analysis: 3191 in the 1970 and 3614 in the 1958 cohort.^2^ A binary outcome variable distinguishing women experiencing natural menopause before age 45 (206 in the 1970 and 368 in the 1958 cohort) from those who experienced natural menopause at 45 or more years, or were peri- or pre-menopause (2985 in the 1970 and 3246 in the 1958 cohort) was defined (Fig. 1A, B). Women with absent menstrual periods due to surgery (331 in the 1970 and 535 in the 1958 cohort) or other reasons (272 in the 1970 and 197 in the 1958 cohort) or who started HT before their FMP (176 in the 1970 and 542 in the 1958 cohort) were excluded. Details about the classification of women into menopause status are provided in Supplementary material (Additional file 1: Tables 1 and 2).

### Potential risk factors

The choice of potential risk factors is guided by the literature reviewed above. Risk factors at birth include: father’s social class, maternal smoking during pregnancy, birthweight standardized for gestational age, maternal age, and breastfeeding. Apart from birth weight and gestational age, which were recorded by a midwife, circumstances at birth were reported by mothers of cohort women. Childhood risk factors include: cognitive ability at age 10 in BCS70 or 11 in NCDS (assessed by reading comprehension and mathematics test scores), age at menarche, and a set of health behaviour characteristics at age 16 – smoking, alcohol consumption, frequency of physical activity, BMI, and psychological distress (assessed by response to 3 items of the Rutter behaviour scale: often worried, miserable, fearful [80]). At ages 10 or 11, reading comprehension and mathematics tests were conducted with cohort members [81, 82]. At 16, information on health behaviour (smoking, drinking and exercise) was provided by cohort women themselves; information about menarche and emotional distress was reported by mothers; and measures of weight and height (used to derive childhood BMI) were recorded at medical examinations. Adulthood risk factors, provided by cohort women, include: the same set of health behaviours – smoking, alcohol consumption, physical activity, BMI, and psychological distress (assessed by response to 24 items of the Rutter Malaise inventory [80]) - measured at age 30 in BCS70 or 33 in NCDS. Other adult risk factors include a set of reproductive characteristics such as experience of period problems or other gynaecological issues by age 30 in BCS70 or 33 in NCDS, use of contraceptives by age 30 in BCS70 or 42 in NCDS, and live births by age 38 in BCS70 or 42 in NCDS; as well as a measure of occupational social class at 38 in BCS70 or 42 in NCDS. When not used as exposures, we control for (some of) these factors as potential confounders (see Statistical analysis). Table 1 details the measures used in this pooled analysis, following data harmonisation. Where possible, we aimed to include exposure variables that precede the onset of the menopause so that the temporal ordering of a potential risk factor and the outcome was not compromised. This, however, cannot be ensured with measures in women’s late thirties or early forties which were included in this analysis due to unavailability of such marker in earlier survey.

**Table 1:** Questions and response options in BCS70 and NCDS and new variables created following data harmonisation

1970 BCS cohort			1958 NCDS cohort			Harmonised measures	
Age (Respondent)	Question	Categories or scale	Age (Respondent)	Question	Categories or scale	Variable	Categories or scale
Birth (midwife)	Birthweight of baby Gestational age	...lbs. ...ozs. or ...gm ...days/...months	Birth (midwife)	Birth weight Gestation period	...lbs. ...ozs. ...days/...months	Birthweight (kg) for gestation (weeks) z-scores ^a^	1st quarter (lowest) 2nd quarter 3rd quarter 4th quarter (highest)
Birth (mother)	Mother’s age at birth	...years	Birth (mother)	Mother’s age at birth	... years	Mother’s age at birth	19 or less years 20–34 years 35 or more years
Birth (mother)	Does the mother smoke now? If no: Did she ever smoke? If yes: How long ago did she stop? How much did she smoke? Has she smoked during this pregnancy?	Yes/No Yes/No Years/Months 1–4/5–14/15–24/25 or more/Not known Yes/No/Not known	Birth (mother)	Did the patient smoke as many as one cigarette a day during the 12 months before the start of this pregnancy? If so, how many per day during that period? If smoked one per day: Did the patient change her smoking habits during this pregnancy? Record changes in the table below and month of pregnancy change made.	Did not smoke as many as 1 per day Number smoked per day in that period No change Gave up/Month of pregnancy/Number per day smoked after change Cut down/Month of pregnancy/Number per day smoked after change Increased/Month of pregnancy/Number per day smoked after change	Mother smoked during pregnancy	No Stopped prior or during the pregnancy Smoked during the pregnancy
Birth (mother)	Occupation of husband If self-employed: If not self-employed: Employed at present Unemployed at present	Actual job, Description of job Employing 25 or more persons/Employing fewer than 25 persons Supervising others/Not supervising others	Birth (mother)	What was the husband’s occupation? If self-employed: Does he employ 10 or more persons? If not self-employed: Does he supervise others (e.g. foreman, manager, charge-hand)?	Actual job / Industry Yes/No Yes/No	Father’s social class at birth	Non-manual Manual No father in household
Birth (mother)	Was {cohort member} breastfed partly or wholly even for a few days?	Yes - for less than 1 month Yes - for 1 month or more but less than 3 months Yes - for 3 months or more Yes but cannot remember for how long No, was not breastfed at all Not known	Birth (mother)	Was the child breastfed (partly or wholly) as a baby?	No Yes - under 1 month Yes - over 1 month Don’t know	Has been breastfed	Less than 1 month or never Breastfed 1 or more months
10 (cohort member)	Friendly Maths Test: A multiple choice test including arithmetic, number skills, fractions, algebra, geometry, and statistics. The Shortened Edinburgh Reading Test (Godfrey Thompson Unit 1978): A test of word recognition, which examined vocabulary, syntax, sequencing, comprehension and retention.	Test scores ranged between 1 and 72 (included 72 items) Test scores ranged between 0 and 67 (included 67 items)	11 (cohort member)	Arithmetic/Mathematics Test: involving numerical and geometric work. Reading Comprehension Test: involving selection of words which appropriately completed sentences	Test scores ranged between 1 and 40 (included 40 items) Test scores ranged between 0 and 35 (included 35 items)	Cognitive ability (maths, reading) z-scores at 10/11 ^b^	… z- score
16 (mother)	What age did your teenage girl have her first menstrual period?	Before 11th birthday When aged 11 Aged 12 Aged 13 Aged 14 Aged 15 or more Not yet commenced Commenced but don’t know age	16 (mother)	At what age did she have her first menstrual period?	Before 11th birthday When aged 11 Aged 12 Aged 13 Aged 14 Aged 15 or more Not yet commenced Commenced but don’t know age Don’t know whether commenced	Age at menarche (years)	… years
16 (medical examination)	Height in cm, to nearest 0.5 cm Height in feet and inches, to nearest 1/4 in. Weight in kilograms, to nearest 0.1 kg Weight in pounds and ounces	… cms … feet … inches … kg … pound … ounces	16 (medical examination)	Height in cm Height in feet and inches Weight	… cm … ft. … in … kg … st … lb.	BMI at 16 (weight [kg]/height [m]2)	… kg/m^2^
16 (cohort member)	Which of the following most nearly describes you?	I have never smoked a cigarette I have only ever tried smoking once or twice I used to smoke sometimes, but I don’t know I smoke and I would like to give it up I do not want to give up smoking	16 (cohort member)	How many cigarettes do you usually smoke in a week?	None, don’t smoke Less than 1 a week Between 1 and 9 a week Between 10 and 19 a week Between 20 and 29 a week Between 30 and 39 a week Between 40 and 49 a week Between 50 and 59 a week 60 or more a week	Has smoked at 16	No Yes
16 (cohort member)	If you have had any alcoholic drink since this time last week, on how many days did you do so?	… number of days	16 (cohort member)	How long is it since you had an alcoholic drink (beer, wine, spirits, etc.)?	Less than 1 week 2–4 weeks 5–8 weeks 9–12 weeks Over 12 weeks Uncertain/Can’t remember Never had one	Has had an alcoholic drink the week prior the age 16 interview	No Yes
16 (cohort member)	During the past year, which of the following {team/individual/other listed} sports did you play, in school and out of school, when they were in season, and how often?	At least once a week At least once a month	16 (cohort member)	Below is a list of things which many people do in their spare time. You will probably only do a few of these. Please show by ringing one of the numbers for each one whether this is something that you do often, sometimes, never or hardly ever. If it is something that you would like to do but don’t have the chance, please ring 4. - Playing outdoor games and sports - Swimming - Playing indoor games and sports	Often Sometimes Never or hardly ever Like to but no chance	Frequency of exercise at 16	Monthly or less often Weekly (or often)
16 (mother)	Below is a series of descriptions of behaviour sometimes shown by young people. Please say whether, in respect of your teenager, the descriptions certainly applies, applies somewhat of doesn’t apply. - Often worried, worries about many things - Often appears miserable, unhappy, tearful or distressed - Tends to be fearful or afraid of new things or new situations	Doesn’t apply Applies somewhat Certainly applies	16 (mother)	Below are a series of descriptions of behaviour often shown by young people. Please ask the informant about each one and ring the appropriate number to show the degree to which this description is true of the study child. - Often worried, worries about many things - Often appears miserable, unhappy, tearful, or distressed - Tend to be fearful or afraid of new things or new situations	Doesn’t apply Applies somewhat Certainly applies	Emotional/Neurotic score at 16 (range: 0–6) ^c^	… score (0 = none apply / 6 = all apply)
30 (cohort member)	How tall are you without shoes? Before you were pregnant, what was your weight? / What is your current weight without clothes on?	Meters and Centimetres Feet and inches Kilograms Stones and pounds	33 (cohort member)	How tall are you without shoes? Before you were pregnant, what was your weight? / What is your current weight without clothes on?	Meters and Centimetres Feet and inches Kilograms Stones and pounds	BMI at 30/33 (weight [kg]/height [m]2)	… kg/m^2^
30 (cohort member)	Would you say that..?	You’ve never smoked cigarettes You used to smoke cigarettes but don’t at all now You now smoke cigarettes occasionally but not every day You smoke cigarettes every day	33 (cohort member)	Do you smoke cigarettes at all nowadays? Have you ever smoked cigarettes regularly - by regularly I mean at least one cigarette a day for 12 months or more?	Yes No Yes No	Has smoked at 30/33	Never Ex-smoker Smoker
30 (cohort member)	How often do you have an alcoholic drink of any kind? Would you say you had a drink..?	On most days 2 to 3 days a week Once a week 2 to 3 times a month Less often or only on special occasions Never now a days Have never had an alcoholic drink	33 (cohort member)	How often do you have an alcoholic drink of any kind?	Most days 1, 2 or 3 times a week 1, 2 of 3 times a month Less often/only on special occasions Never	Frequency of alcohol consumption at 30/33	Weekly (once to most days a week) Monthly (one to three times a month) Less often or never
30 (cohort member)	Do you regularly take part in any of the activities on this card - that is at least once month, for most of the year? How often do you take part in any activity of this type?	Yes No Every day or most days 4–5 days a week 2–3 days a week Once a week 2–3 times a month Less often	33 (cohort member)	Do you regularly take part in any of the activities on this card - that is at least once month, for most of the year? How often do you take part in any activity of this type?	Yes No Can’t say Every day or most days 4–5 days a week 2–3 days a week Once a week 2–3 times a month Less often Can’t say	Frequency of exercise at 30/33	Weekly (once to most days per week) Monthly (two to three times a month) Less often or never
30 (cohort member)	Malaise inventory, 24-items measuring psychological distress	Yes No	33 (cohort member)	Malaise inventory, 24-items measuring psychological distress	Yes No	Has experienced depression symptoms at 30/33 ^d^	No (score 0–7) Yes (score of 8 or higher)
30 (cohort member)	Have you ever had or been told that you had a problem with your periods? Have you ever been told that you had any other gynaecological problems?	Yes No Yes No	33 (cohort member)	Have you ever suffered from or been told you had..? - Persistent trouble with periods - Other gynaecological problems	Yes Only when pregnant No	Has suffered periods or other gynaecological problems by 30/33	No Yes
30 (cohort member)	Are you currently taking the contraceptive pill? Have you ever taken the contraceptive pill?	Yes No Yes No	42 (cohort member)	Are you currently taking the contraceptive pill? Have you ever taken the contraceptive pill?	Yes No Yes No	Has ever taken contraceptive pill by 30/42	No Yes
33, 38 (cohort member)	Has anyone you were having a sexual relationship with ever become pregnant? / have you been pregnant? What was the result of this pregnancy [for the nth baby]?	Yes No Live birth Still birth Miscarriage Abortion Still pregnant	33, 42 (cohort member)	Has anyone you were having a sexual relationship with ever become pregnant? / have you been pregnant? What was the result of this pregnancy [for the nth baby]?	Yes No Live birth Still birth Miscarriage Abortion Still pregnant	Has no live born children (nulliparous)	No Yes
38 (cohort member)	Which of the things on this card best describes what you were doing {date of last interview}{next}? What is your (main) job? What do you mainly do in your job? What does the firm or organisation you work for mainly make or do (at the place where you work)?	Full-time paid employee (30 or more hours a week) Part-time paid employee (under 30 hours a week) Full-time self-employed Part-time self-employed Unemployed and seeking work Full-time education On a government scheme for employment training Temporarily sick/disabled Permanently sick/disabled Looking after home/family Wholly retired Other (specify) Open text Open text Open text	42 (cohort member)	Which of the things on this card best describes what you are currently doing? What is your (main) job? What do you mainly do in your job? What does the firm or organisation you work for mainly make or do (at the place where you work)?	Full-time paid employee (30 or more hours a week) Part-time paid employee (under 30 hours a week) Full-time self-employed Part-time self-employed Unemployed and seeking work Full-time education On a government scheme for employment training Temporarily sick/disabled Permanently sick/disabled Looking after home/family Wholly retired Other (specify) Open text Open text Open text	Social class at 38/42	Non-manual Manual Not in paid employment

^a^Birthweight was standardized by gestational age as the difference between the individual’s birthweight (in kg) and the mean birthweight for that gestational age (in weeks) was divided by the standard deviation of birthweight for that gestational age (in weeks) [27]. Gestational age was categorized into six groups: < 37 weeks, 37, 38, 39, 40, > 40 weeks. Birthweight for gestation z-scores was subsequently categorized into quarters (lowest to highest)

^b^Single main component (factor) score for cognitive ability (using maths and reading tests at age 10 in BCS and age 11 in NCDS) was extracted using Principal Components Analysis (PCA). The scores were standardised to a mean of zero and a standard deviation of one [81]

^c^Psychological distress in childhood was derived by summing responses given to the ‘fearful’, ‘miserable’ and ‘worried’ items, loading on the Rutter emotional-neurotic factor [80]. Items were measured on a 3-category scale from 0 to 2, where ‘0 = Does not apply’, ‘1 = Applies somewhat’, and ‘2 = Certainly applies’. Score range from 0 to 6; a high score indicates emotional problems

^d^Psychological distress in adulthood was derived by summing responses given to the 24 ‘yes-no‘items of the Rutter Malaise inventory [80]. Scores range from 0 to 24; a score of 8 or higher is considered a cut off for experiencing symptoms consistent with depression and used here to derive a binary measure (presence or absence of depression symptoms) [83]

### Statistical analysis

Descriptive statistics for all variables included in the analysis for each cohort separately, including means and standard deviation, median and range for continuous variables, and percentages with 95% confidence intervals for categorical variables, are presented in Supplementary material (Additional file 1: Table 3).

We use multivariable logistic regression and present adjusted odds ratios and 95% confidence intervals for the association between each potential risk factor and menopause before 45 years after controlling for confounders. The data for the two cohorts are pooled with models incorporating a dummy variable identifying which cohort the woman belonged to (1958 or 1970). We use multiple imputation techniques to tackle data missingness (see Missing data). Respecting the temporal sequence of exposures, we fit a series of models to estimate associations of risk factors adjusted for preceding variables only. We estimate the association between early menopause and: social class at birth controlling for cohort year only; maternal smoking controlling for social class; birthweight (standardized for gestational age) controlling for social class, maternal smoking, and maternal age at birth; breastfeeding controlling for all other birth variables. We estimate the association between early menopause and cognition (at age 10 or 11) controlling for all birth factors. We then fit a model including all other childhood risk factors, controlling for cognitive ability and birth factors.^3^ Finally, a model was used to estimate the associations between early menopause and all adulthood risk factors, controlling for childhood and birth factors. We also fit models for each potential risk factor adjusted for cohort year only and the results are presented in Supplementary material (Additional file 1: Table 4).

The pooled cohort analysis enables us to estimate associations with increased statistical power (due to the increased size of our analytical sample) and focus on the common factors affecting menopause before 45 (rather than associations observed in each study separately). The sequential regression modelling, adjusted for preceding factors only (as confounders) and not mediators, aims to estimate (adjusted) associations which may reflect causal effects (under assumptions of no unmeasured confounding or other sources of bias) [84, 85].

In secondary analyses, we carried out logistic regression analyses relating menopause before 45 to the birth, childhood, and early adulthood variables, in each cohort separately. These results are presented in Supplementary material (Additional file 1: Tables 5 and 6).

All analyses were conducted using Stata version 15.0.

### Missing data

To avoid the limitation of performing this analysis on a significantly reduced analytical sample, due to simultaneous inclusion of exposure variables from different periods of life and with different levels of unit and item missingness, we used Multiple Imputation (MI) with chained equations [86–88], under an assumption of missing at random (MAR),^4^ performed in each cohort separately. The proportion of missing observations in potential risk factors range between 4 to 31% in NCDS and 3 to 57% in BCS70 (Additional file 1: Table 3). Further to being of substantive interest in this study, previous research on predictors of non-response in the 1970 and 1958 cohorts has shown that birth and early life characteristics, including social class at birth, maternal age at birth, breastfeeding, childhood cognition and mental health, are important determinants of non-response in the cohorts and can make the MAR assumption more plausible. Respectively, including these variables in the imputation models can help reduce bias due to missing data and restore sample representativeness [86–89]. The imputation model included all potential risk factors (exposures) and menopause status (outcome), but missing values were only imputed on exposures (see details about Menopause status missingness in Menopause status and in Supplementary material (Additional file 1: Tables 2.1 and 2.2)) [88]. Fifty imputed datasets were created for each cohort, in line with the recommendation that the number of imputations should be comparable to the proportion of missing data [90], and models were subsequently fitted using the combined (1970 and 1958 cohort) imputed datasets. The imputation was conducted using Stata version 15.0.

## Results

The analytical sample comprised 6805 (natural) menopausal, peri- and pre- menopausal women (3191 participating in the 1970 and 3614 participating in the 1958 cohort). By age 45, 206 (6.5, 95% CI: 5.7–7.4) women in BCS70 and 368 (10.2, 95% CI: 9.2–11.2) women in NCDS analytical samples had undergone natural menopause. The pooled sample comprised of 574 (8.4, 95% CI: 7.8–9.1) early menopausal women. Women’s birth, childhood, and adult characteristics, in each cohort separately, are presented in Supplementary material (Additional file 1: Table 3).

### Birth models

Table 2 reports associations between women’s traits at birth and the subsequent probability of early menopause. Women whose fathers at birth worked in manual occupations had a twofold increase in the odds (2.0 times higher) for early menopause compared to women whose fathers occupied non-manual jobs; the increase in the odds was more than twofold (2.2 times higher) for women whose fathers were absent at the time of their birth. Women whose mothers smoked during pregnancy had 24% higher odds for early menopause compared to women whose mothers did not smoke during pregnancy. Women who were breastfed for less than a month had 30% higher odds for early menopause compared to women who were breastfed for 1 month or longer. Younger age of mother at birth was associated with increased odds for being early menopausal, but the 95% confidence interval included 1. There was no evidence for association between birthweight (standardized for gestational age) and early menopause.

**Table 2:** Odds Ratios (OR) and 95% confidence intervals (CI) for being early menopausal on birth characteristics (*n* = 6805)

Birth characteristics	OR	95% CI	
**Father’s social class**^a^			
No father in household/Other	2.20***	1.48	3.28
Manual	2.02***	1.61	2.54
Non-manual (reference)	1		
**Mother smoked during pregnancy**^b^			
No (reference)	1		
Stopped prior or during the pregnancy	0.77	0.50	1.18
Smoked during pregnancy	1.24*	1.03	1.49
**Maternal age at birth**^c^			
19 or less years	1.25	0.90	1.74
20–34 years (reference)	1		
35 or more years	1.13	0.85	1.48
**Birthweight standardized for gestation**^d^			
1st quarter (lowest)	1.01	0.78	1.30
2nd quarter	0.91	0.70	1.18
3rd quarter (reference)	1		
4th quarter (highest)	1.05	0.80	1.39
**Breastfed**^e^			
Less than 1 month	1.30*	1.05	1.60
1 or more months (reference)	1		

This table presents selected coefficients for being early menopausal on birth characteristics. Full results are presented in Supplementary material (Additional file 1: Table 4)

An asterisk indicates significance levels, *** *p* < 0.001, * *p* < 0.05

Adjusted for:

^a^ Cohort year

^b^ Father’s social class, and cohort year

^c^ Father’s social class, maternal smoking during pregnancy, birthweight standardized for gestational age, and cohort year

^d^ Father’s social class, maternal smoking during pregnancy, maternal age at birth, and cohort year

^e^ Father’s social class, maternal smoking during pregnancy, maternal age at birth, birthweight standardized for gestational age, and cohort year

### Childhood models

Table 3 presents associations between childhood characteristics and early menopause. The odds for early menopause decreased with an increase in childhood cognitive ability. One standard deviation higher cognitive ability score corresponded to 36% lower odds for being early menopausal. Women who smoked at 16, compared to those who did not, had 51% higher odds for early menopause.

**Table 3:** Odds Ratios (OR) and 95% confidence intervals (CI) for being early menopausal on childhood characteristics (*n* = 6805)

Childhood characteristics	OR	95% CI	
**Cognitive ability at 10/11 (per SD)**^a^	0.64***	0.57	0.71
**Age at menarche (per year)**	0.98	0.90	1.07
**BMI at 16 (per kg/m2)**	1.03	0.99	1.07
**Smoking at 16**			
No (reference)	1		
Yes	1.51***	1.19	1.92
**Drinking the week prior age 16 interview**			
No (reference)	1		
Yes	1.09	0.85	1.39
**Exercise at 16**			
Monthly or less often (reference)	1		
Weekly (Often)	0.92	0.70	1.20
**Emotional/Neurotic score at 16**	1.00	0.92	1.10

The second childhood model (from which all the other coefficients come from) included all remaining potential risk factors in childhood (age at menarche, BMI, smoking, alcohol drinking, exercise, and emotional/neurotic score), adjusted for cognitive ability at 10/11 and birth characteristics, and cohort year

This table presents selected coefficients for being early menopausal on childhood characteristics. Full results are presented in Supplementary material (Additional file 1: Table 4)

An asterisk indicates significance levels, *** *p* < 0.001

^a^ Adjusted for all birth characteristics, and cohort year

There was no evidence that menarcheal age, alcohol drinking, exercise, or emotional problems at 16, were associated with menopause before 45.

### Early adulthood model

Table 4 presents associations between characteristics recorded in women’s early adulthood, and their probability of early menopause. Women who experienced problems with periods or other gynaecological issues by their early thirties, and those who smoked in their early thirties, were 68 and 69% more likely to be menopausal before 45, respectively. On the other hand, alcohol drinking, once to three times per month, compared to less frequent consumption, was associated with decreased odds for early cessation of menstrual periods, although there was no evidence of a trend across categories. Further, the odds for early menopause decreased with one or more days of exercise per week compared to less frequent exercise. Women who were not in paid employment in their late thirties or early forties were more likely to undergo early menopause. This includes women who were unemployed, in education, sick or disabled, or looking after home or family.

**Table 4:** Odds Ratios (OR) and 95% confidence intervals (CI) for being early menopausal on early adulthood characteristics (*n* = 6805)

Early adulthood characteristics	OR	95% CI	
**BMI at 30/33 (per kg/m2)**	0.99	0.96	1.02
**Smoking at 30/33**			
Never (reference)	1		
Ex-smoker	1.03	0.77	1.39
Smoker	1.69***	1.28	2.23
**Alcohol consumption at 30/33**			
Less often or never (reference)	1		
Monthly (one to three times a month)	0.76**	0.57	1.00
Weekly (once to most days a week)	0.84	0.67	1.06
**Exercise at 30/33**			
Less often or never (reference)	1		
Monthly (two to three times a month)	1.18	0.79	1.76
Weekly (once to most days a week)	0.75***	0.60	0.93
**Depression symptoms at 30/33**			
No (reference)	1		
Yes	1.11	0.83	1.48
**Periods or other gynaecological problems by 30/33**			
No (reference)	1		
Yes	1.68***	1.36	2.06
**Used oral contraceptives by 30/42**			
No (reference)	1		
Yes	0.85	0.63	1.16
**No live born children by 38/42 (nulliparous)**			
No (reference)	1		
Yes	1.13	0.88	1.45
**Social class at age 38/42**			
Not in paid employment	1.43***	1.13	1.81
Manual	0.96	0.74	1.24
Non-manual (reference)	1		

The early adulthood model included all potential risk factors in adulthood, adjusted for all childhood and birth characteristics, and cohort year

This table presents selected coefficients for being early menopausal on early adulthood characteristics. Full results are presented in Supplementary material (Additional file 1: Table 4)

An asterisk indicates significance levels, *** *p* < 0.001, ** *p* < 0.01

Other factors, such as BMI, mental health status, use of oral contraceptives or nulliparity were not significantly associated with early menopause.

## Discussion

We found that multiple factors from birth, childhood and adulthood are associated with menopause before 45 years of age. The early life factors associated with early menopause were father in a manual job, or no father figure at birth, mother smoking during pregnancy, and the absence of or short duration of breastfeeding. Childhood factors increasing the likelihood of early menopause were poor cognitive ability and smoking. Early adulthood factors associated with an increased probability of early menopause were smoking, no alcohol consumption, lower levels of exercise, problems with periods or other gynaecological issues, and not being in paid work.

Our findings support the hypothesis that fetal and early life experiences are associated with age at menopause. Consistent with previous studies, we find socioeconomic position, captured here by father’s social class at birth, to be related to the timing of menopause. The mechanism underlying this association is unclear but may act through health behaviour such as diet and nutrition or may be due to its relationship with early emotional stress, for example that caused by the absence of a parent or other adverse experiences as suggested by previous studies [20, 38].

Our findings on the role of maternal smoking are consistent with the hypothesis that prenatal exposure to cigarette smoking may supress the formation of the ovarian follicle pool or damage it [32]. Our results on an association between breastfeeding and early menopause are consistent with those from the NSHD [23], described previously with the role of nutrition [13, 30, 31]. Inconsistencies with other studies showing no association [25] may be due to retrospective reporting of breastfeeding in such studies or factors controlled for in the analyses.

Our observation of a strong association with childhood cognitive ability is consistent with findings from other British birth cohort studies [39], where “genetic programming” has been suggested as a possible explanation for this link (or that same or overlapping genetic mechanisms may be determining the neurocognitive function and the permanent cessation of menstrual periods) [15, 17].

Our study adds to the already consistent evidence that cigarette smoking is associated with early menopause. We also support previous findings that earlier initiation of smoking is linked to earlier menopause [57] as we find increased odds of early menopause with smoking at age 16 years.

Smoking was not the only health-related behaviour associated with the timing of menopause, however, physical activity and alcohol consumption were associated with lower odds for being early menopausal. Our analysis suggests that it is high levels of activity, in a woman’s early thirties, one to several days of exercise a week, which is associated with lower odds for early menopause. Previous research on the role of physical activity on menopause is scarce and inconclusive, however it has been demonstrated that women’s reproductive hormones can be affected by physical factors [52]. We found that drinking, one to three times a month, in a woman’s early thirties, compared to less frequent drinking, lowered the odds for early menopause, but that there was no difference in odds for heavier drinkers. Although this observation is in line with a study using UK Biobank data [25], previous research on alcohol has not been consistent. Some studies have suggested that moderate alcohol consumption increases oestrogen levels in women [50, 51]. However, other studies have argued that health benefits of light to moderate alcohol use, but not heavier, are likely to be spurious and due to control group misclassification (e.g., some abstainers may be former dependent or heavy drinkers, who quit because of poor health, or individuals with long-term chronic illness) and residual confounding [91].

Gynaecological health was strongly linked to early menopause in our study. Previous evidence on the impact of gynaecological health on menopause timing is scarce. Gynaecological problems were not associated with early menopause in a study using UK Biobank data [25] but have been linked to it indirectly in studies on (in) fertility [47]. As different gynaecological conditions may have different effects on menopause, exploring the role of specific gynaecological problems (e.g., polycystic ovary syndrome (PCOS), endometriosis, or sexually transmitted infections) is important to better understand the relationship with menopause timing.

Contrary to the hypothesis [92] our results do not indicate an association between age of menarche or live born children with the timing of menopause (in either crude or adjusted models). Noting the wide range of factors our models accounted for (in both childhood and adulthood), including other markers of reproductive health, this may suggest that menarche or parity effects observed previously might be due to residual confounding or recall error [93]. Age at menarche in our analyses is reported by mothers when daughters were aged 16 rather than self-reported in adulthood [94]; and nulliparity derived using pregnancy histories through the study lifecycle.

And lastly, we observed that women not in paid employment in their late thirties or early forties had greater odds of early menopause. Previous findings have not been consistent including a study using NCDS data (up until age 45) which found no effect on menopause before 45 years [27]. This, again, may be indicative of the importance of the timing of risk factor exposure. We note, that in our study this measure is not strictly preceding the menopause but is likely representative of earlier adult socioeconomic position.

There are limitations to consider in our study. As with any longitudinal study, both cohorts have been affected by attrition. Fortunately, the retention rates over the decades following the study members have been strong [86]. The proportion of women lost to follow-up (due to permanent refusal, inability to trace, emigration, or death) by age 42 survey in the 1970 cohort and age 44/45 survey in the 1958 cohort (when questions about menopause were first asked), was approximately a quarter of the original sample in both cohorts. A further 5% percent were lost to follow-up in the subsequent sweep (age 46 in the 1970 cohort and age 50 in the 1958 cohort). Participation rates in these follow-up sweeps varied between 71 and 81% (of all eligible women). Other factors that affected the size of our analytical sample were insufficient information to derive menopause status (in some circumstances due to survey error^5^) and the exclusion of women based on the definition of our target population.

Other limitations include the retrospective collection of information on menopause and potential for recall bias although recall was not over a long period. Previous research has indicated that menopause is reasonably well recalled but the recall error is expected to increase with the time after menopause^6^ [95]. Additionally, the recall of multiple events (e.g., amenorrhea, surgery, and HT initiation) and the associated dates may be more susceptible to recall error (we assign menopause status based on the first experienced event). Another possibility for misclassification comes from the use of menstrual bleeding criteria to assign menopause status (rather than hormonal levels) which may be problematic in the presence of conditions that affect the menstrual bleeding (e.g., abnormal menstrual patterns following endometrial ablation, presence of polycystic ovarian syndrome, or other uterine or ovarian anomalies, including excessive exercise).

We investigated the role of a large number of potential risk factors at different stages of life, and with different levels of (unit and item) missingness. We noted missingness in risk factors between 4 and 31% in the 1958 and 3–57% in the 1970 cohort (Additional file 1: Table 3). We approached the missing data problem with MI using a high number of imputations in line with the recommendation [90]. Further, we noted that (birth and early life) characteristics of substantial research interest in our study have been found previously to affect non-response in both cohorts; therefore, including these characteristics in our imputation model and analysis can help reduce bias due to missing data and restore sample representativeness [86, 89]. Despite our efforts, bias due to selective attrition cannot be ruled out.

We include a wide range of prospectively collected risk factors ensuring in all models that known and measured confounders are accounted for, but potential biases resulting from unobserved confounding (compromising the estimation of causal effects in observational studies, alongside other sources of bias) is inevitable. We do not account for the variation in the experience of menopause before 45 years among women of different ethnic backgrounds because both cohorts are of predominantly White ethnic background and very few women (less than 10) of non-White ethnic background have undergone early menopause in our analytical sample.

Our analysis investigates common risk factors for women born 12 years apart which may obscure cohort-specific relationships. We use a combined study because of the comparable study designs and measures but acknowledge the potential for bias in the harmonized data that may result from combining data from different studies (e.g., due to age and periods effects, or different level of sample attrition and missing data) which may in turn affect the generalizability of our findings. Nonetheless, it is reassuring that the associations presented here are consistent with those observed in each cohort separately. If statistical significance is not reached in a cohort, the estimated effects remain in the same direction (see study-specific analyses in Supplementary material: Additional file 1: Tables 5 and 6). As we aim to ensure comparability in the derived measures, potentially important risk factors may have been disregarded if relevant information was only available in one of the cohorts (e.g., specific problems with periods such as heavy periods or gynaecological conditions such as endometriosis which may be associated with different age at menopause). Another possible limitation is the absence of specific measures (e.g., type and dose of contraceptives) in both studies which may affect the observed associations. And finally, our analysis did not consider the possibility that premature (menopause before 40 years of age) and early menopause (between 40 and 44 years) might be affected by distinct (non-genetic) factors. Previous genetic research has questioned whether premature menopause is determined by the same genes as menopause that occurs between 40 and 44 years [14].

## Conclusions

In conclusion, we investigated risk factors for early natural menopause (before the age of 45) in two population-based prospective cohort studies of women born in Britain in 1958 and 1970 and followed from birth to adulthood. We demonstrated that characteristics at different stages of life are associated with menopause before the age of 45 – an event with serious health consequences. Some of these associations may reflect genetic or underlying biological processes which with further investigation could lead to better understanding of the menopausal process and reproductive health across the life course. Further, some of the characteristics associated with early menopause relate to behaviours which are modifiable (e.g., breastfeeding, smoking, physical activity). If the presented associations reflect causal effects, early (timely) prevention may reduce the risks of early menopause and hence the adverse health outcomes associated with it.

## Supplementary Information

**Additional file 1: Supplemetary material.** Risk factors for natural menopause before the age of 45: evidence from two British population-based birth cohort stuides. Harmonised variables used in the analysis (Table 1 Menopause status); classification of women into menopause status (Table 2.1, 2.2 Menopause status); descriptive statistics for all variables included in the analysis for each cohort separately (Table 3 Descriptive statistics); univariate and multivariate study specific (Tables 5,6 Study specific) and pooled (Table 4 Pooled models) regression models.

## Data Availability

BCS70 and NCDS data can be accessed via the UK Data Service (UKDS) [76, 77]. The datasets generated and analysed for this study are available from the corresponding author on reasonable request.

## Abbreviations

BCS70: 1970 British Cohort Study
NCDS: 1958 National Child Development Study
NSHD: 1946 National Survey of Health and Development
BMI: Body mass index
HT: Hormone therapy
FMP: Final menstrual period

## Footnotes

1 Formally, early menopause is defined as menopause between the ages of 40 and 44, and the onset of menopause before age 40 as premature menopause [5, 6].

2 Following further exclusion of twins and triplets and women with missing multiple birth information (182 in the 1970 and 135 in the 1958 cohort). This is because previous research has established strong genetics relationship between being a child of a multiple birth and premature or early menopause [5].

3 The association between age at menarche and early menopause has also been estimated without other age 16 variables (i.e., excluding BMI, smoking, drinking, exercise, and emotional distress), controlling for cognitive ability and factors at birth only. No difference from the presented estimate was observed (results not shown).

4 It implies that systematic differences between the missing and observed data can be explained by the observed data [87].

5 For example, women in the 1970 cohort who at age 42 survey reported amenorrhea due to other (known) reasons (incl. Pregnancy, breastfeeding, contraception) were erroneously omitted the menstrual cycle questions in the subsequent age 46 survey. As a result, menopause status has not been assigned for those women.

6 Due to an error, women in the 1958 cohort who reported natural menopause in the age 44/45 follow-up were not asked about their age at their FMP; they were asked about this subsequently at the age 50 follow-up, which increases the risk for recall error.
